# Simultaneous Reduction and Functionalization of Graphene Oxide by 4-Hydrazinobenzenesulfonic Acid for Polymer Nanocomposites

**DOI:** 10.3390/nano6020029

**Published:** 2016-02-04

**Authors:** Song-Jie Qiao, Xiang-Nan Xu, Yang Qiu, He-Chong Xiao, Yue-Feng Zhu

**Affiliations:** 1 School of Materials Science and Engineering, Tsinghua University, Beijing 100084, China; qiaosongjie19900627@hotmail.com (S.-J.Q.); xvxiangnan@gmail.com (X.-N.X.); 2 Key Laboratory for Advanced Materials Processing Technology, Ministry of Education, Beijing 100084, China; qiuyangdme@hotmail.com (Y.Q.); thudmeonion@gmail.com (H.-C.X.); 3 Department of Mechanical Engineering, Tsinghua University, Beijing 100084, China

**Keywords:** graphene, functionalization, reduction, nanocomposite

## Abstract

Graphene oxide (GO) was functionalized and reduced simultaneously by a new reductant, 4-hydrazinobenzenesulfonic acid (HBS), with a one-step and environmentally friendly process. The hydrophilic sulfonic acid group in HBS was grafted onto the surface of GO through a covalent bond. The successful preparation of HBS reduced GO (HBS-rGO) was testified by scanning electron microscope (SEM), X-ray diffraction (XRD), Raman spectroscopy, Fourier transform infrared spectra (FTIR), X-ray photoelectron spectroscopic (XPS) and thermogravimetric analysis (TGA). The interlayer space of HBS-rGO was increased to 1.478 nm from 0.751 nm for GO, resulting in a subdued Van der Waals’ force between layers and less possibility to form aggregations. The aqueous dispersibility of graphene was improved to 13.49 mg/mL from 0.58 mg/mL after the functionalization. The viscosity of the epoxy resin based HBS-rGO composite could be regulated by an adjustment of the content of HBS-rGO. This study provides a new and applicable approach for the preparation of hydrophilic functionalized graphene, and makes it possible for the application of graphene in some functional polymer nanocomposites, such as specialty water-based coatings.

## 1. Introduction

Graphene, a one-atom-thick planar sheet of sp^2^-hybridized carbon atoms arranged in a two-dimensional lattice, has drawn more and more attention since being found in 2004, because of its excellent electrical and thermal conductivities, superior mechanical properties, high aspect ratio, remarkable chemical inertness, and impermeability to fluids and gases [1,2]. These properties make graphene a viable candidate for some functional polymer nanocomposites, such as specialty coatings designed to have the characteristics of electromagnetic interference (EMI) shielding and corrosion protection [3,4,5]. However, the applications of graphene in environmentally friendly water-based coatings, which are the trend of future coatings, are restricted by the hydrophobic property of graphene [6,7,8].

The chemical functionalization of graphene oxide (GO) has attracted a great deal of attention because of its wide application [9,10,11,12], such as optical limiting property [13], energy conversion [14], biosensing systems [15,16], gas separating membranes [17,18], improved electric property [19,20], enhanced dispersibility [21] and thermal stability [22]. Several chemical functionalization methods designed to improve the dispersion of graphene in water were reported. Cai *et al.* functionalized GO with glycidol, and reduced the functionalized graphene oxide by sodium borohydride [23]. Kuila *et al.* utilized 6-amino-4-hydroxy-2-naphthalenesulfonic acid to functionalize GO, and chose hydrazine monohydrate as reductant [24]. Xu *et al.* prepared stable aqueous dispersions of graphene sheets using 1-pyrenebutyrate and then reduced the functionalized graphene oxide by hydrazine monohydrate [25]. These methods generally involved two steps: functionalizing the graphene oxide by hydrophilic functional groups and then reducing the GO to graphene by certain reductant. However, many vessels and different reaction conditions were used in these two-step methods, which made them unsuitable for mass production [26,27,28]. What is worse is, the most commonly used reductants to restore the sp^2^ structure of graphene are anhydrous hydrazine, hydrazine monohydrate, sodium borohydride, and hydrogen sulfide, which are highly toxic and harmful to both living organisms and the environment [29,30,31,32]. Thus, surface functionalization and milder reduction of GO are desirable.

In this study, GO was functionalized and reduced simultaneously by 4-hydrazinobenzenesulfonic acid (HBS). Two functional groups are included in HBS, which are the sulfonic acid group and hydrazine group. The sulfonic acid group, which is hydrophilic, was grafted onto the surface of GO through a covalent bond, and oxygen-containing groups such as hydroxy and carboxyl were reduced by the hydrazine group at the same time. Moreover, HBS is hardly toxic to the human body. Therefore, this one-step method is easier to operate, achieves better mass production than the two-step one, and is more friendly to the environment. The successful reduction and functionalization of GO by HBS was verified by several characterization methods. The epoxy based HBS reduced GO (HBS-rGO) nanocomposites were prepared and their viscosity behavior was verified. This study makes it possible to compound graphene with the hydrophilic matrix, and has broad application prospects in specialty water-based coatings.

## 2. Experimental

### 2.1. Materials

HBS was purchased from Sigma-Adrich (Steinheim, Germany). Graphene and Graphite oxide fine powder were supplied from Nanjing XF NANO Materials Tech Co., Ltd., Nanjing, China. Epoxy was purchased from Nantong Xingchen Synthetic Material Co., Ltd., Nantong, China. All other chemical materials were purchased from Sigma-Adrich and used as received.

### 2.2. Materials Synthesis

**Figure 1:**
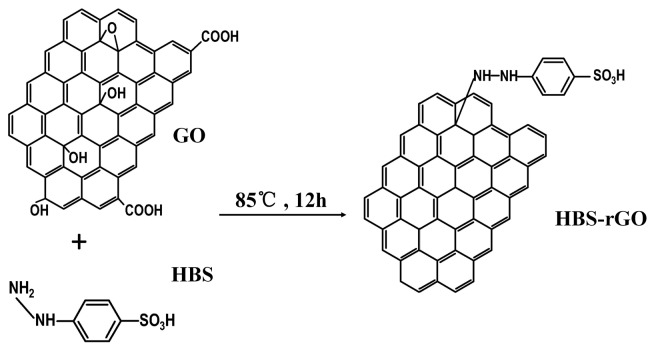
Reaction scheme for preparation of 4-hydrazinobenzenesulfonic acid (HBS) reduced graphene oxide (GO) (HBS-rGO).

By the epoxy ring-opening reaction, graphene surface was grafted with HBS layer with hydroxyl groups. The preparation procedure of HBS-rGO is described in Figure 1. First, 0.3 g of GO powder and 1.2 g of HBS were added into 100 mL distilled water. After being kept under ultrasound (45 kHz, 100 W) for 20 min and stirred for 5 min, the mixture was heated at 85 °C for 12 h. The black graphene product was dried and purified using 50% ethanol to completely remove the residual impurities.

The epoxy based HBS-rGO composite was prepared by solution mixing method [33,34,35]. The desired amount of HBS-rGO was first dispersed in ethanol via ultrasonication for 2 h. The calculated amount of epoxy resin was dissolved in ethanol under constant stirring. The dispersion of HBS-rGO was then added to the epoxy resin solution with vigorous stirring. Finally, the composite solution was dried in a vacuum oven for 48 h at 80 °C. The weight percentage of HBS-rGO/epoxy composite was determined to be 0.50 wt %, 0.75 wt % and 1.00 wt %, respectively. The 0.50 wt % graphene/epoxy and 0.50 wt % GO/epoxy composites were prepared by the same method.

### 2.3. Materials Characterizations

The functionalization and reduction of graphene oxide by HBS was characterized by SEM, XRD, Raman spectroscopy, FTIR, XPS spectra and TGA. SEM images were taken by ZEISS MERLIN Compact (Oberkochen, Germany). XRD tests were conducted on Rigaku Corporation SmartLab (Tokyo, Japan). The Raman spectra were excited with a laser of 488 nm and recorded on solid powder samples using a LabRAM HR800 spectrometer (Paris, France). FTIR spectra were collected on a Perkin-Elmer spectrometer (Norwalk, CT, USA) using KBr pellets. XPS measurements were performed on Thermo Fisher ESCALAB 250Xi (Maple Plain, MN, USA). TGA measurements were carried out on a Q5000 TGA of TA instruments (New Castle, DE, USA) at a heating rate of 10 °C/min from 30 °C to 700 °C in nitrogen.

## 3. Results and Discussion

### 3.1. SEM Analysis of GO and HBS-rGO

**Figure 2:**
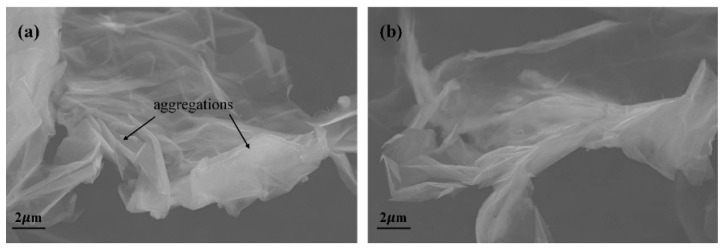
Scanning electron microscope (SEM) images of (**a**) GO and (**b**) HBS-rGO.

Morphology of the GO and HBS-rGO samples are characterized by SEM. As shown in Figure 2, the morphology of the samples exhibited layered structures. The samples of GO were found to be more likely to form aggregations than HBS-rGO (indicated by the arrows in Figure 2), as a result of shorter interlayer space and stronger Van der Waals’ force. This result was also confirmed by the XRD results.

### 3.2. X-Ray Diffraction Measurements of GO and HBS-rGO

**Figure 3:**
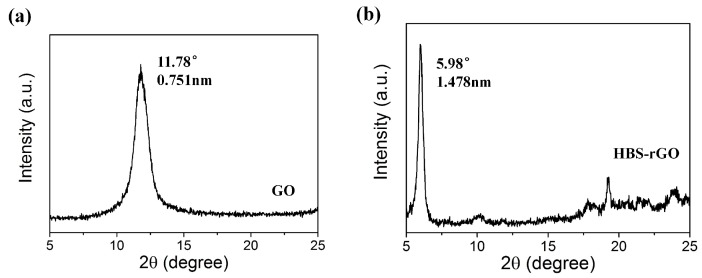
The X-ray diffraction (XRD) patterns of (**a**) GO and (**b**) HBS-rGO.

The XRD patterns of GO and HBS-rGO are shown in Figure 3. The broad band for GO was at 2θ = 11.78, corresponding to an interlayer space of approximately 0.751 nm, which was in good agreement with previous results [36,37]. The broad band for HBS-rGO was downshifted to 2θ = 5.98, corresponding to an interlayer space of 1.478 nm. The significant increase in the interlayer space indicated further exfoliation of the graphene sheets causedg by the HBS branches on the surface of GO. This result manifested the GO was functionalized by HBS. The Van der Waals’ force between the layers got weaker with increased interlayer space, and this made HBS-rGO less likely to form aggregations, which agreed with the results of SEM analysis.

### 3.3. Raman Spectroscopy of GO, HBS-rGO and Graphene

**Figure 4:**
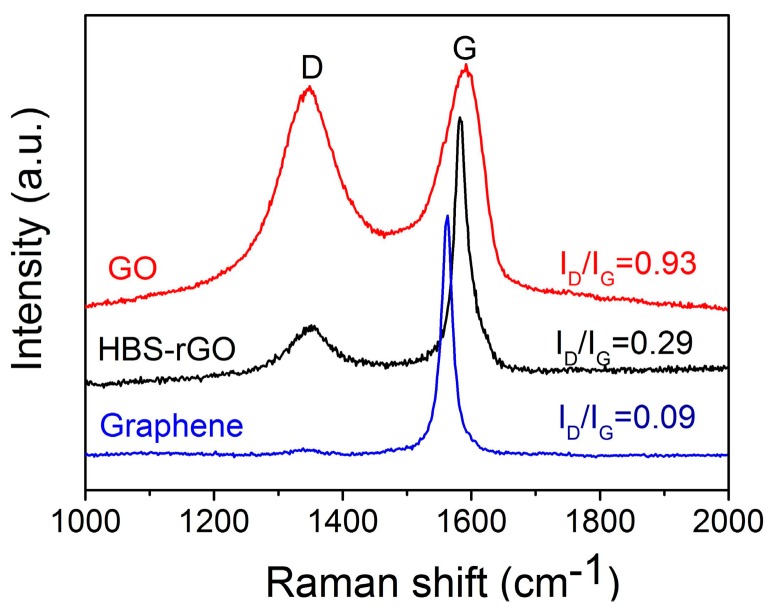
The Raman spectroscopy of GO, HBS-rGO and grapheme.

The Raman spectroscopy results of GO, HBS-rGO and graphene are shown in Figure 4. Two obvious bands located at around 1590 cm^−1^ and 1350 cm^−1^ were observed, which were generally assigned as the D band (at about 1350 cm^−1^) and G band (at about 1590 cm^−1^), corresponding to the structural defects and vibration of sp^2^-hybridized graphitic domains, respectively [38,39]. The intensity ratio of the two bands (I_D_/I_G_) proved the graphitization degree of carbon solids and a lower value represented a higher degree of graphitization. The ratio of I_D_/I_G_ of HBS-rGO was 0.29, which was much smaller than that of GO and very close to the value of graphene. Meanwhile, the G peak of HBS-rGO was red-shifted to 1582 cm^−1^, getting close to that of graphene (1563 cm^−1^), implying the restoration of the graphitic sp^2^ network. These results clearly indicated that the GO was well reduced by HBS.

### 3.4. FTIR Spectroscopy of GO and HBS-rGO

**Figure 5:**
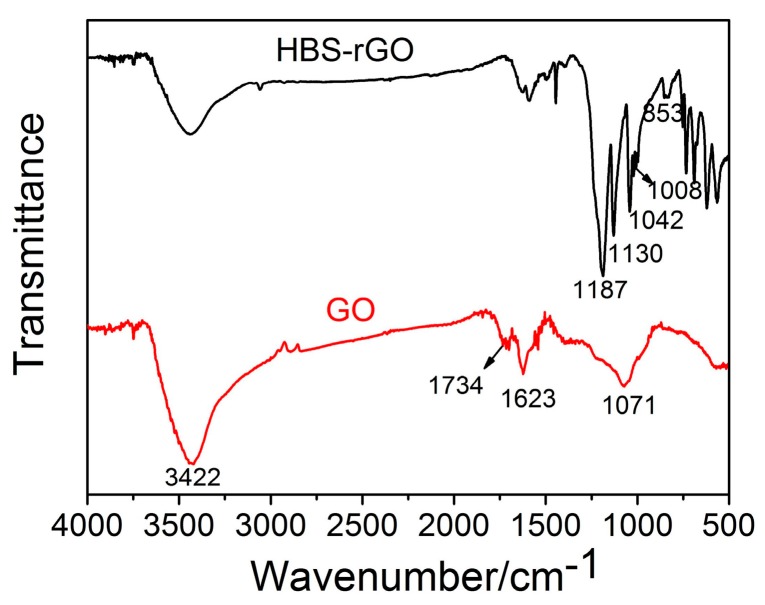
The Fourier transform infrared spectra (FTIR) spectra of GO and HBS-rGO.

Figure 5 exhibits the Fourier transform infrared (FTIR) spectra of GO and HBS-rGO. The characteristic peaks of GO were at 3422 (C–OH), 1734 (C=O), 1623 (C=C), and 1071 cm^−1^ (C–O) [40,41]. These peaks became significantly weaker for HBS-rGO, which indicated GO was greatly reduced by HBS. The deformation vibration for S=O at 1187 cm^−1^, S–O at 1130 cm^−1^ and S-phenyl at 1042 cm^−1^ in HBS-rGO appeared and confirmed the presence of sulfonic groups. Besides, C–H at 1008 and 853 cm^−1^ in HBS-rGO showed that the benzene ring in HBS was para-substituted by –NH and –SO_3_H. These results indicated GO was successfully reduced and functionalized by HBS.

### 3.5. XPS Spectroscopy of GO and HBS-rGO

**Figure 6:**
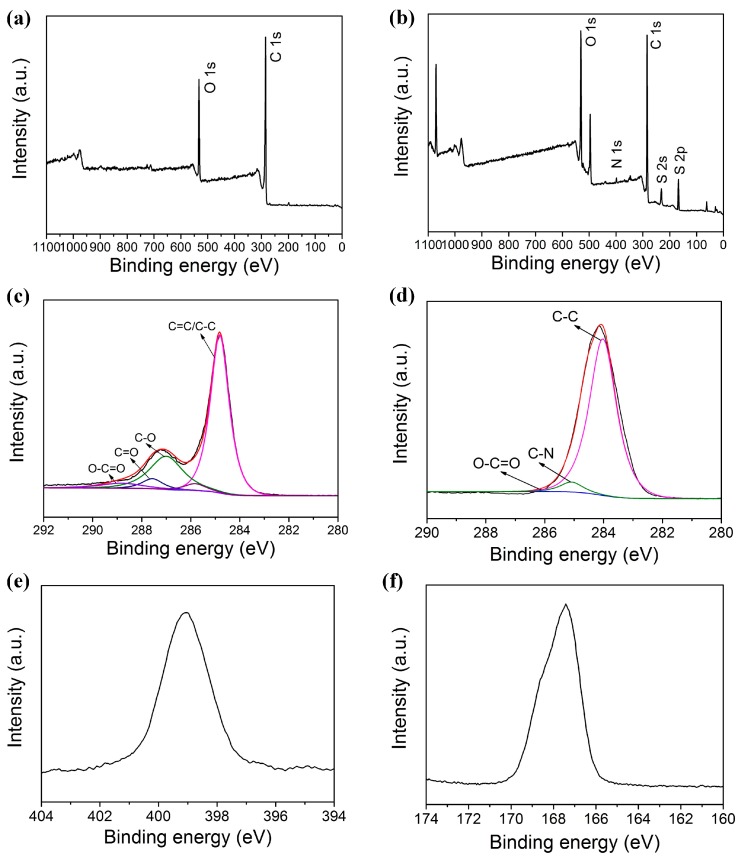
The X-ray photoelectron spectroscopic (XPS) spectra of GO and HBS-rGO: all elements survey scan of GO (**a**), HBS-rGO (**b**), C 1s survey scan of GO (**c**), HBS-rGO (**d**), (**e**) N 1s, and (**f**) S 2p for HBS-rGO.

The XPS spectra of GO and HBS-rGO are shown in Figure 6. The contents of C and O elements in GO were 82.81% and 17.19%, respectively. While the contents of C, O, N and S elements in HBS-rGO were 67.34%, 21.33%, 1.92% and 9.4%, respectively. Considering that –SO_3_H was the main form of S element, the O content in other oxygen-containing groups of HBS-rGO was 7.23%. The dropping of O content from 17.19% for GO to 7.23% for HBS-rGO (except the O in –SO_3_H group) was owing to the successful reduction of GO by HBS. Figure 6c,d shows the C 1s spectra of GO and HBS-rGO, respectively. As can be seen, the intensity of the C–O (286.9 eV) and C=O (287.6 eV) in GO both decreased significantly after the reduction. Besides, the N–H group of hydrazine group was shown in the N 1s XPS spectrum in Figure 6e, whose BE was 399.1 eV. The S 2p XPS spectrum of HBS-rGO in Figure 6f showed that the BE was 167.5 eV, which was bigger than elemental S 2p (165 eV). This result confirmed that –SO_3_H was the main form of S element. These results demonstrated that GO was successfully reduced by HBS and the hydrazine group, and sulfonate group was successfully grafted onto the graphene.

### 3.6. TGA Measurements of GO and HBS-rGO

**Figure 7:**
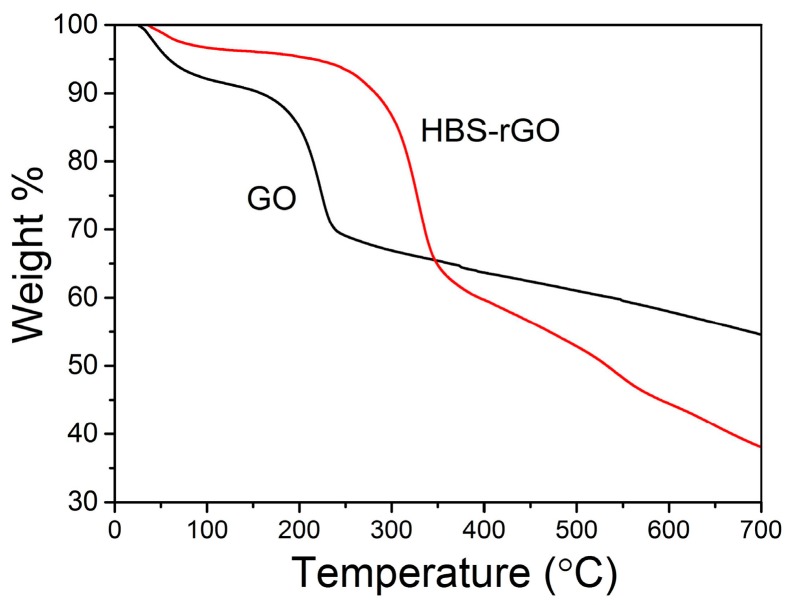
Thermogravimetric analysis (TGA) measurements of GO and HBS-rGO.

TGA measurement provides further proof of the reduction of GO by the functionalization of HBS. As shown in Figure 7, for GO, the mass loss was about 10% before 100 °C, owing to the removal of adsorbed water. The mass loss at around 200 °C was about 30%, which was attributed to the decomposition of labile oxygen functional groups. For HBS-rGO, only 5% of the main mass loss was appeared at around 200 °C. This result indicated that no more than 5% of the labile oxygen functional groups were left in the HBS-rGO. Therefore, the TGA measurement of GO and HBS-rGO confirmed the successful reduction of GO by the functionalization of HBS.

### 3.7. Aqueous Dispersibility of HBS-rGO

**Figure 8:**
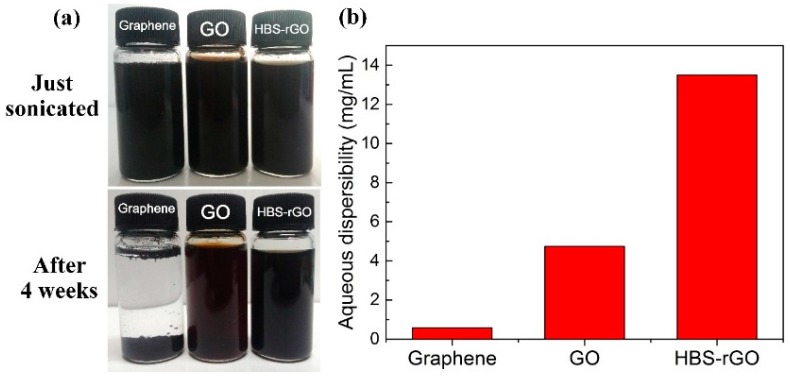
(**a**) Digital images of graphene, GO and HBS-rGO aqueous solutions (1.5 mg/mL) after being kept for four weeks. (**b**) Aqueous dispersibility of graphene, GO and HBS-rGO.

The dispersibility of graphene in water was greatly improved after the functionalization due to the emergence of the hydrophilic sulfonic acid group. The aqueous solution of graphene formed sediments in less than 30 s, while a uniformly dispersed 1.5 mg/mL aqueous solution of HBS-rGO could be kept for weeks without any extra additives, as shown in Figure 8a. The dispersibility of HBS-rGO in water was measured by following method and the results are shown in Figure 8b [42]. One gram HBS-rGO was added into 50 mL distilled water and homogenously dispersed by sonication for 1 h. The resulting dispersion of HBS-rGO was centrifuged at 3000 rpm for 30 min. Then, 20 mL of the upper supernatant suspension was carefully taken and dried at 80 °C in vacuum for 12 h, at which point it was weighed to determine dispersibility. The experiment was carried out threee times and the average of the results was taken as the final dispersibility of HBS-rGO. The dispersibility of graphene and GO in water was measured by the same method. The aqueous dispersibility of HBS-rGO was measured to be 13.49 mg/mL, which was much better than the aqueous dispersibility of graphene (0.58 mg/mL) and GO (4.74 mg/mL), as a result of successful functionalization of hydrophilic sulfonic acid group. Meanwhile, the Van der Waals’ force between the HBS-rGO layers became weaker than graphene and GO with increased interlayer space, which made HBS-rGO less likely to form aggregations and possess much better aqueous dispersibility.

### 3.8. Viscosity Behavior of HBS-rGO/epoxy Composite

**Figure 9:**
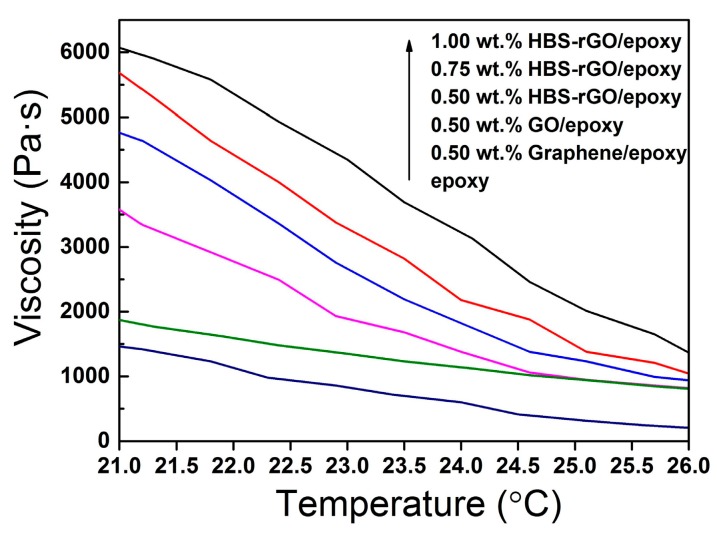
The viscosity behavior of epoxy resin based composites.

In order to verify the viscosity behavior of epoxy resin based HBS-rGO composite, graphene, GO and HBS-rGO/epoxy nanocomposites with certain contents of filler were prepared by solution mixing, and the relation between viscosity and temperature was studied. The weight percentage of graphene/epoxy and GO/epoxy composite were both 0.50 wt %. The weight percentage of HBS-rGO/epoxy composite was determined to be 0.50 wt %, 0.75 wt % and 1.00 wt %, respectively. The results are shown in Figure 9. The viscosity of epoxy resin based composites decreased rapidly with the increase of temperature from 21 °C to 26 °C. In the low temperature zone, the viscosity of 0.50 wt % HBS-rGO/epoxy composite (about 3600 Pa∙s at 21 °C) was much higher than that of GO/epoxy (about 1800 Pa∙s at 21 °C) and graphene/epoxy composite (about 1500 Pa∙s at 21 °C). This was because the molecular space structure of graphene and GO changed from two-dimensional to three-dimensional after being functionalized by HBS, and the intermolecular forces between graphene and epoxy resin matrix were significantly reinforced by the branches of HBS on the surface of graphene. Meanwhile, HBS-rGO was less likely to form aggregations and dispersed much better than graphene and GO in the epoxy matrix as a result of bigger interlayer space and weaker Van der Waals’ force. In the high temperature zone, the viscosity of epoxy resin based composites was much lower and became closer to the viscosity of epoxy because the space between the molecules of composites became larger and the intermolecular forces decreased with the increase of temperature. Besides, the viscosity of composites increases with the increased content of HBS-rGO in composites. According to this phenomenon, the viscosity of the epoxy resin based HBS-rGO composite can be well regulated by an adjustment of the content of HBS-rGO.

## 4. Conclusions

In summary, graphene oxide was functionalized and reduced simultaneously by a new reductant, 4-hydrazinobenzenesulfonic acid, with a one-step and environmentally friendly process. The hydrophilic sulfonic acid group in HBS was grafted onto the surface of GO through a covalent bond. The successful functionalization and reduction was testified by SEM, XRD, Raman spectroscopy, FTIR, XPS and TGA. The interlayer space of HBS-rGO was increased to 1.478 nm from 0.751 nm for GO, resulting in the subdued Van der Waals’ force between layers and less possibility to form aggregations. The O content dropped from 17.19% for GO to 7.23% for HBS-rGO (except the O in –SO_3_H group) owing to the successful reduction of GO by HBS. The aqueous dispersibility of graphene was improved to 13.49 mg/mL from 0.58 mg/mL after the functionalization. The viscosity of the epoxy resin based HBS-rGO composite can be regulated by an adjustment of the content of HBS-rGO. This study shows HBS-rGO is a potential filler material for some functional polymer nanocomposites, such as specialty water-based coatings.
